# Effect of Pressure on Ce-Substituted Nd-Fe-B Hot-Deformed Magnets in the Hot-Pressing Process

**DOI:** 10.3390/ma17153769

**Published:** 2024-07-31

**Authors:** Ye Ryeong Jang, Wonjin Kim, Sumin Kim, Wooyoung Lee

**Affiliations:** 1 Department of Materials Science and Engineering, Yonsei University, Seoul 03722, Republic of Korea; j_ye_r@yonsei.ac.kr (Y.R.J.); clsrn0926@gmail.com (W.K.); 2 Department of Magnetic Materials, Korea Institute of Materials Science, Changwon 51508, Republic of Korea

**Keywords:** Nd-Fe-B permanent magnets, hot deformed magnet, Ce-substituted magnet, microstructure

## Abstract

With the increasing demand for Nd-Fe-B magnets across various applications, the cost-effective substitution of Ce has garnered significant interest. Many studies have been conducted to achieve the high magnetic properties of Nd-Ce-Fe-B hot deformation magnets in which Nd is replaced with Ce. We propose a method to improve magnetic properties of the Ce-substituted Nd-Ce-Fe-B hot-deformed magnets by optimizing the hot-pressing process. This study investigates the microstructure and properties following hot deformation of Ce-substituted Nd-Ce-Fe-B magnets fabricated at a constant temperature and different pressures (100–300 MPa) during the hot-pressing process. The results highlight the influence of pressure from previous hot-pressing processes on grain alignment and microstructure during hot deformation. Magnets subjected to hot pressing at 200 MPa followed by hot deformation achieved superior magnetic properties, with H_ci_ = 8.9 kOe, B_r_ = 12.2 kG, and (BH)_max_ = 31 MGOe with 40% of Nd replaced with Ce. Conversely, precursors prepared at 100 MPa exhibited low density due to high porosity, resulting in poor microstructure and magnetic properties after hot deformation. In magnets using precursors prepared at 300 MPa, coarsened grains and a condensed h-RE_2_O_3_ phase were observed. Incorporating Ce into the magnets led to insufficient formation of RE-rich phases due to the emergence of REFe_2_ secondary phases, disrupting grain alignment and hindering the homogeneous distribution of the RE-rich phase essential for texture formation. Precursors prepared under suitable pressure exhibited uniform distribution of the RE-rich phase, enhancing grain alignment along the c-axis and improving magnetic properties, particularly remanence. In conclusion, our findings present a strategy for achieving the ideal microstructure and magnetic properties of hot-deformed magnets with high Ce contents.

## 1. Introduction

Nd-Fe-B permanent magnets, known for their outstanding magnetic properties, are widely employed in high-performance motors such as those found in battery generators and electric vehicles. The rapid market growth has significantly increased the demand for Nd-Fe-B magnets, leading to heightened risks of price volatility and supply instability for rare-earth elements like Nd and Pr [1,2]. Consequently, extensive research is being conducted to develop permanent magnets that reduce or eliminate the need for Nd, using the more abundant and cost-competitive Ce [3,4,5,6,7,8,9]. However, the magnetic properties of Ce-substituted magnets are significantly inferior to those of traditional Nd-Fe-B magnets because Ce_2_Fe_14_B (saturation magnetization (J_s_) = 11.7 kG, anisotropy field (H_A_) = 26 kOe, Curie temperature (T_c_) = 151 °C) have inferior intrinsic magnetic characteristics and thermal stability compared to Nd_2_Fe_14_B (J_s_ = 16.0 kG, H_A_ = 73 kOe, T_c_ = 312 °C) [10,11].

The hot deformation process has proven to be an efficient technique for obtaining high-density anisotropic magnets with nanocrystalline grains. This method shows potential for improving the low magnetic anisotropy of Ce_2_Fe_14_B and enhancing coercivity by finely tuning the grain size to the single domain level [4,12]. Pathak et al. first demonstrated that coercivity could be improved through the formation of nanocrystalline grains and the application of the die upset process in composition where 20% of Nd is replaced with Ce [13]. In this method, melt-spun ribbons containing randomly oriented Nd_2_Fe_14_B nanograins undergo hot pressing to form a dense isotropic body. This body is then subjected to hot deformation, resulting in anisotropic magnets with a platelet-like textured structure. These platelet Nd_2_Fe_14_B grains range in thickness from 50 to 150 nm, with their c-axis aligned parallel to the pressing direction [14,15].

However, achieving high magnetic properties in hot-deformed magnets with a high level of Ce substitution remains challenging. In the Ce-Fe-B alloy phase diagram, the appearance of secondary phases such as CeFe_2_ and Ce_2_Fe_17_ prevents the formation of well-defined Ce-rich grain boundary phases compared to Nd-Fe-B [16]. Huang et al. enhanced the magnetic properties by adding intergranular Pr-Cu to improve plastic deformation and c-axis orientation. Additionally, Tang et al. improved the coercivity of Ce hot-deformed magnets by enhancing the grain boundary phase through the diffusion of a Nd-Cu eutectic alloy [4,17]. On the other hand, when the Ce substitution level exceeds the 24 wt.%, a significant amount of the CeFe_2_ phase forms, which has a melting point of 925 °C [7,18,19,20], higher than the temperature used in the hot deformation process. This leads to the presence of precipitates during hot deformation, disrupting grain alignment and significantly reducing coercivity and remanence [21]. Recently, Lee et al. reported the coercivity of 15 kOe and remanence of 13 kG for Ce-substituted (Nd_0.7_Ce_0.3_)–Fe–B hot-deformed magnets without REFe_2_ phases using amorphous ribbons [21]. By suppressing the precipitation of the REFe_2_ phase and increasing the volume fraction of the RE-rich liquid phase, the c-axis alignment of the 2:14:1 platelet was improved.

Various studies have been conducted to improve the magnetic properties of Ce-substituted hot-deformed magnets by suppressing secondary phases and enhancing the grain boundary. Our focus is on optimizing the microstructure of the hot-pressed precursor as a method to suppress secondary phases and improve grain boundaries. One of the mechanisms driving grain alignment is the anisotropic grain growth that occurs as the grain boundaries phase melts during hot-pressing. This is followed by the promotion of crystal rotation through grain boundary sliding within the c-plane [22]. Therefore, during the hot-pressing step, a uniformly formed RE-rich grain boundary phase with high wettability is necessary to enhance the c-axis crystallographic orientation through grain boundary sliding [23]. Several studies have reported that the magnetic properties of hot-deformed magnets vary with changes in pressure or temperature conditions during the hot-pressing process [24,25]. However, the effects of magnetic properties and microstructural changes during the intermediate hot-pressing step in Ce-substituted hot-deformed magnets have not been clearly reported. Thus, in this work, we investigated the influence of hot-pressing pressure on magnetic and microstructural properties of the final hot-deformed magnet. In particular, we analyzed the changes in the magnetic properties, microstructures, and texture of the magnets upon the hot-press step and hot-deformation step, respectively. Based on the results obtained, we propose a method to improve the magnetic properties of the Ce-substituted hot-deformed magnets.

## 2. Materials and Methods

Alloys with a composition of Nd_18.2_Ce_11.81_Co_3.6_Ga_0.53_Fe_64.9_B_0.92_ (wt.%, Nd:Ce = 3:2) were prepared via arc melting of pure metals (99.9 wt.%). The resulting ingot was then melt-spun into amorphous ribbons at a wheel speed of 35 m/s under an argon atmosphere. The diameter of the quartz nozzle used for melt-spinning was 0.38 mm. The ribbons were subsequently crushed into flakes with particle sizes ranging from 100 to 300 µm using a mortar. The flakes were hot-pressed in a tungsten carbide mold with diameter of 10.5 mm at 700 °C under various pressures (100, 200, and 300 MPa) for 20 min in a vacuum. The hot-pressed precursors, designated as HP100, HP200, and HP300 for those prepared at 100, 200, and 300 MPa, respectively, underwent hot deformation at 780 °C with a deformation rate of 0.004 s^−1^ and deformation degree, ε, of 1.2 [ε = ln(h_0_/h), h_0_: height of the samples before deformation, h: height of the samples after deformation], which corresponds to a 70% height reduction. The resulting hot-deformed magnets were labeled as HP100-HD, HP200-HD, and HP300-HD, respectively.

The density was measured using Analytical Balance (METTLER TOLEDO-MR204, Columbus, OH, USA,) and the magnetic properties were evaluated using a B-H tracer (Permagraph C-300, Magnet Physik, Köln, Germany). Magnetic hysteresis loops were measured on a cylindrical sample with a diameter of 24 mm and height of 2 mm. The phases in the samples were characterized by X-ray diffraction (XRD, Rigaku, Tokyo, Japan) with Cu-Kα radiation (1.5418 Å, 40 kV, 40 mA). The microstructure was examined by scanning electron microscopy (SEM, JEOL-7800F, JEOL Ltd., Tokyo, Japan) and transmission electron microscopy (TEM, JEOL-JEMARM200F, JEOL Ltd., Tokyo, Japan) equipped with an energy-dispersive spectrometer (EDS).

## 3. Results and Discussion

**Figure 1:**
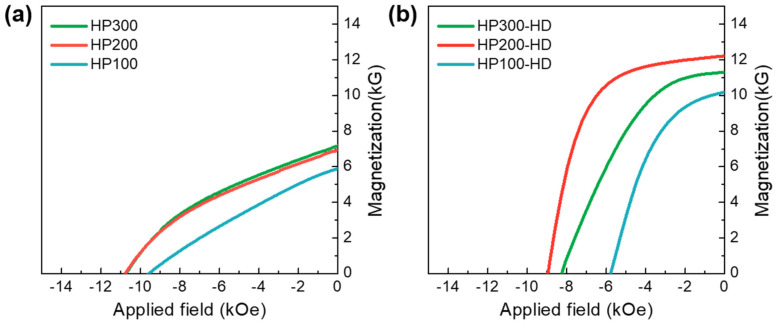
Demagnetization curves for the (**a**) hot−pressed precursors and (**b**) hot−deformed magnets fabricated from precursors pressed at different pressures.

Figure 1a shows the magnetic hysteresis loops measured for hot-pressed precursors prepared under various pressures. The magnetic properties of the isotropic sintered body, specifically remanence (B_r_) and coercivity (H_ci_), exhibited an increase as the pressure escalated from 100 MPa to 300 MPa. The relative density of the sintered precursors obtained through hot-pressing at 100 MPa was only 89%, as shown in Table 1. However, precursors hot-pressed at 200 and 300 MPa demonstrated a significantly higher relative density, reaching up to 98%. The elevated pressure in the hot-pressing step contributed to an increase in the density and volume of the crystalline hard magnetic RE_2_Fe_14_B phases, thereby enhancing magnetic properties [24]. Despite having the same density, there were observable differences in the magnetic properties of HP200 and HP300 after undergoing hot deformation. The magnetic properties of final (Nd_0.6_Ce_0.4_)–Fe-B hot-deformed magnets are presented in Figure 1b and Table 1. After hot deformation, all specimens exhibited an increase in remanence and a decrease in coercivity.

**Figure 2:**
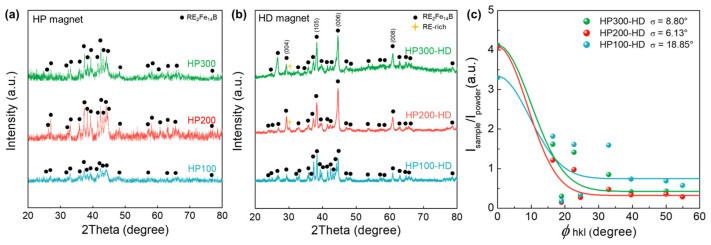
XRD patterns of the (**a**) hot-pressed precursors and (**b**) hot-deformed precursors. (**c**) Gaussian-fitted curves for the relative intensity versus angle between the c-axis and normal (h k l) in the RE_2_Fe_14_B crystal for the hot-deformed magnets.

Figure 2a shows the XRD patterns collected on the surface perpendicular to the pressing direction of the hot-pressed magnets obtained under varying pressures. The amorphous melt-spun flake gradually crystallized during hot pressing, resulting in discernible peaks of the RE_2_Fe_14_B main phase in HP100, HP200, and HP300. However, a relatively low peak intensity of HP100 implies that it contains amorphous flakes that remained uncrystallized. Elevated pressure increases sample density, improving not only the contact area between the sample and the mold but also promoting greater contact among the constituent particles of the compacts resulting from pressing. This facilitates crystallization by expediting heat transfer and a subsequent temperature rise of the particles [26]. The diffraction peaks for each specimen after hot deformation are shown in Figure 2b. The peaks of the RE-rich phase are evident in both HP200-HD and HP300-HD, with higher intensity in HP200-HD. The observed RE-rich phase diffraction peak is attributed to the formation of a RE-rich grain boundary phase. The HP100-HD sample produced under lower pressure conditions than HP200,300-HD exhibited low density and insufficient crystallization during the hot-pressing step due to inadequate heat transfer. Consequently, additional crystallization occurred during the subsequent hot deformation process. Therefore, the intensity of the (0 0 6) peak for HP100-HD is lower than that of the hot-deformed magnets prepared at high pressures during hot pressing. The peak intensity ratio of the (0 0 6) to (1 0 5) planes, R(0 0 6)/(1 0 5), is a common metric for characterizing the c-texture in hot-deformed magnets [27]. The Gaussian standard deviation, calculated as the angle between the easy axis and magnetization direction based on the XRD pattern measured in the c-plane [28], is presented in Figure 2c. A smaller standard deviation, σ, suggests the improved c-axis alignment of the magnets. The vertical values represent the distribution of the relative intensity of RE_2_Fe_14_B, while the horizontal values indicate the difference in the angle between the c-axis and the (h k l) normal to the RE_2_Fe_14_B crystal. HP100-HD, lacking c-axis alignment, is close to isotropic and has the largest σ value of 18.85°. Meanwhile, the σ values for HP200-HD and HP300-HD are 6.13° and 8.80°, respectively, aligning with the trend of their remanence values (Figure 1b and Table 1). These results indicate that the grain alignment and c-axis orientation of the final hot deformed magnet are influenced by crystallization and anisotropic grain growth during the hot-pressing step, resulting in distinct outcomes. Therefore, pressure variations during the hot-pressing process can determine the microstructure of the sintered body and significantly impact the final texture of the magnet. In the Ce-substituted magnets, the insufficient presence of RE-rich grain boundaries results from the stable formation of the REFe_2_ secondary phase. This contributes to a significant decrease in magnetic properties when the Ce content exceeds a specific threshold [29,30,31,32]. Lee et al. reported coercivity of 15 kOe and remanence of 13 kG for Ce-substituted (Nd_0.7_Ce_0.3_)–Fe–B hot-deformed magnets without REFe_2_ phases using amorphous flakes [21]. In our study, no diffraction peaks of the REFe_2_ phase are observed in Figure 2a,b, indicating that the use of amorphous melt-spun ribbon effectively suppresses the formation of the REFe_2_ phase, even in the composition where 40 wt.% of Ce replaced Nd, surpassing 30 wt.%.

**Figure 3:**
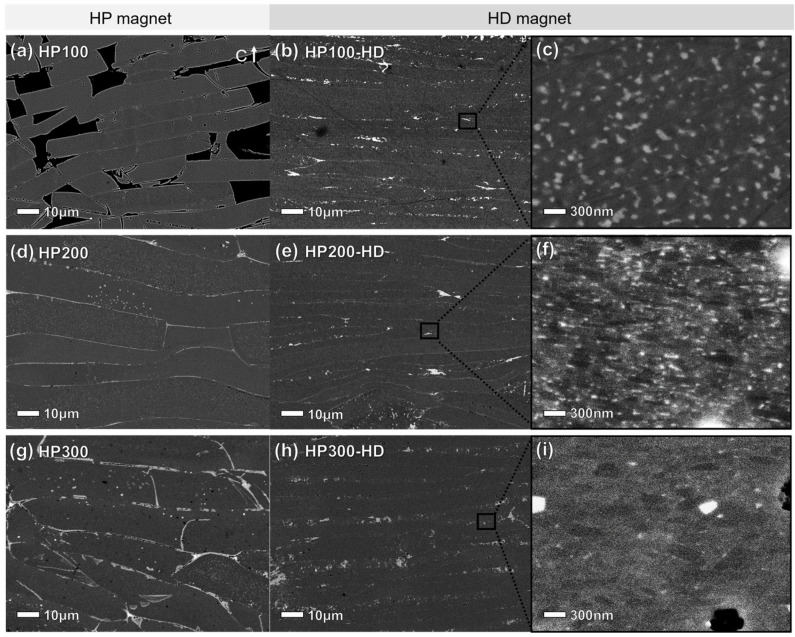
BSE SEM images of the hot-pressed precursors prepared at different pressures (**a**) HP100, (**d**) HP200, and (**g**) HP300; and hot-deformed magnets (**b**,**c**) HP100-HD, (**e**,**f**) HP200-HD, and (**h**,**i**) HP300-HD at low and high magnifications, respectively.

Figure 3 shows cross-sectional images of hot-pressed precursors and hot-deformed magnets using backscattered electron (BSE) scanning electron microscopy (SEM) at various pressures. In HP100 (Figure 3a), numerous pores are observed at the boundaries of ribbon flakes, explaining the low relative density in Table 1. Figure 3b shows microstructures after deformation, and Figure 3c provides a high magnification image of the ribbon flake boundary. Following hot pressing, hot deformation caused the RE-rich phase to fill the gap between flakes, resulting in only compression without significant anisotropic grain growth. As depicted in Figure 3c, the grains assumed nearly circular shapes with minimal uniaxial alignment. This indicates that the low pressure during hot pressing hindered the growth into platelet shapes, impacting c-axis alignment even after hot deformation. Figure 3d depicts the distribution of uniform RE-rich phase along the flake boundary in HP200. After hot deformation, as shown in Figure 3e, the RE-rich phase is thinner and more evenly distributed between the compressed flakes compared to HP100-HD. A high-magnification image of the flake boundary (Figure 3f) reveals platelet-shaped grains stacked along the c-axis, with interspersed RE-rich phase. The uniformly distributed RE-rich phase at the flake boundaries promotes anisotropic grain growth and deformation through grain boundary sliding. In contrast, HP300 exhibits a non-uniform and thick RE-rich phase at the flake boundary, as shown in Figure 3g. The agglomeration of the RE-rich phase at the flake boundary resulted in excessive liquid supply, accelerating grain growth [33]. These distributions of RE-rich phases persisted as agglomeration at the flake boundary during subsequent hot deformation processes (Figure 3h). As demonstrated in Figure 3i, due to the low melting point, applied pressure during hot deformation led to the squeezing out of the RE-rich phase, transitioning into a precipitated phase between grains [34]. Consequently, larger misaligned grains are observed in HP300-HD (Figure 3i) compared to HP200-HD (Figure 3f). The different pressures applied during the hot-pressing step impacted not only the grain size but also the distribution of the grain boundary phase in the final hot-deformed magnets.

**Figure 4:**
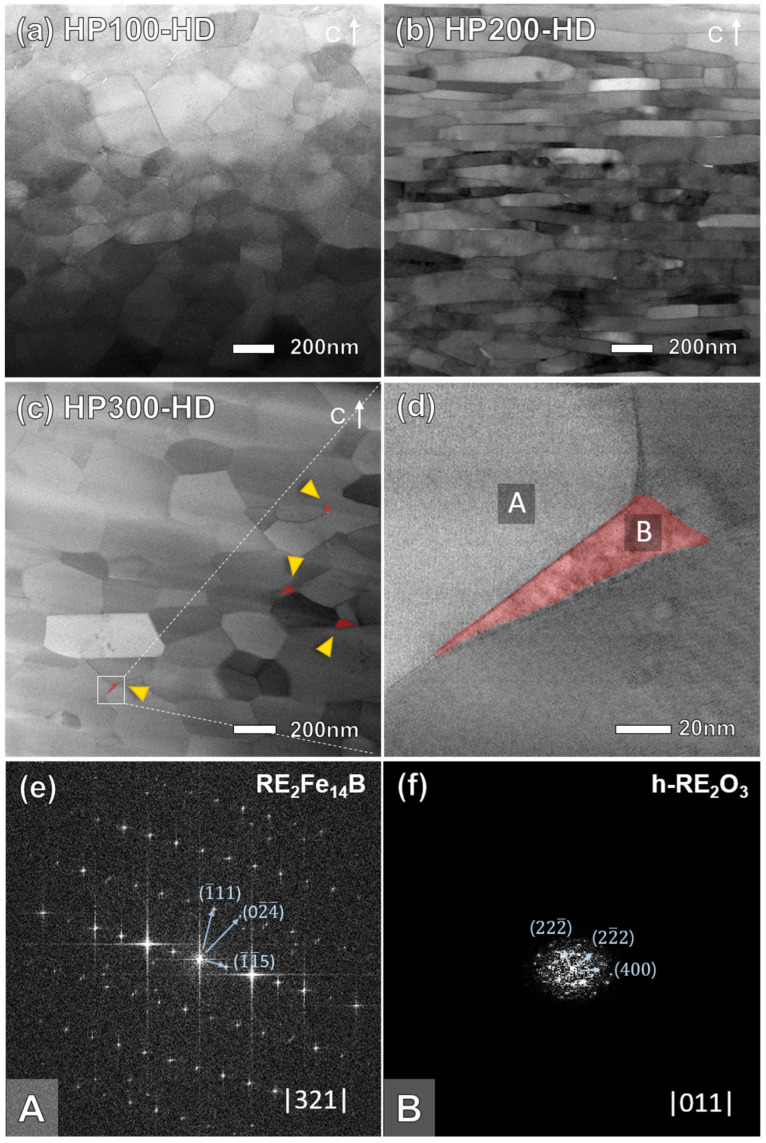
ABF-STEM images of (**a**) HP100-HD, (**b**) HP200-HD, (**c**) HP300-HD, and (**d**) the region marked by the white box in (**c**). FFT patterns of the selected area marked with (**e**) A (RE_2_Fe_14_B main phase) and (**f**) B (precipitated h-RE_2_O_3_ phase) in (**d**).

Figure 4a–c show annular bright-field scanning transmission electron microscopy (ABF-STEM) images at the flake boundaries of HP(100,200,300)-HD, respectively. In HP100-HD (Figure 4a), misaligned fine nanocrystalline grains with a size of 200–300 nm are observed instead of plate-like grains growing along the a- or b-axis, consistent with the XRD analysis results and BSE images described previously. In contrast, HP200-HD exhibits thin and densely stacked plate-shaped grains, aligned along the c-axis, with an average width and height of 300–400 and 30–50 nm, as shown in Figure 4b. In Figure 4c, HP300-HD reveals partially observed plate-shaped grains. However, most of the grains are randomly oriented like HP100-HD, and larger coarse grains compared to HP100-HD are observed. Excessive pressure during the hot-pressing step causes aggregation of the RE-rich phase at the flake boundary, providing a diffusion path for elements and promoting grain growth [25]. Moreover, precipitated secondary phases are prominently observed in HP300-HD in the regions marked by the red areas between grains. Fast Fourier Transform (FFT) analysis in the areas marked as A and B in Figure 4d identifies these phases as RE_2_Fe_14_B and hcp-RE_2_O_3_ (a = 0.388 nm, c = 0.604 nm), respectively (h-Nd_2_O_3_, a = 0.383 nm, c = 0.599 nm/h-Ce_2_O_3_, a = 0.389 nm, c = 0.606 nm). These aggregates existed as a stable phase containing a significant amount of oxygen due to RE oxidation. The lumpy RE-rich phase, with a large specific surface area, is prone to oxidation, and due to the higher activity of Ce compared to Nd, a considerable amount of RE-rich oxides exists in the grain boundary region [35,36]. Unlike fcc-NdO_2_ and c-Nd_2_O_3_, which are grain boundary phases that can increase coercivity by minimizing lattice mismatch, h-Nd_2_O_3_ with an oxygen content of 55 to 70 at.% improves corrosion resistance but weakens magnetic properties, reducing the density and coercivity of the magnet [37]. The agglomerated RE-rich liquid phase during the hot-pressing step precipitated as a h-RE_2_O_3_ phase at the grain boundaries and triple junctions after subsequent deformation processes, significantly reducing the wettability. As a result, RE-rich grain boundaries were not formed uniformly and sufficiently, failing to effectively inhibit magnetic exchange coupling and also reducing grain alignment effects during hot deformation [17,38,39]. Additionally, excessive liquid supply in specific areas promotes grain coarsening, which disrupted grain alignment and decreased magnetic properties in the final hot-deformed magnet [33].

**Figure 5:**
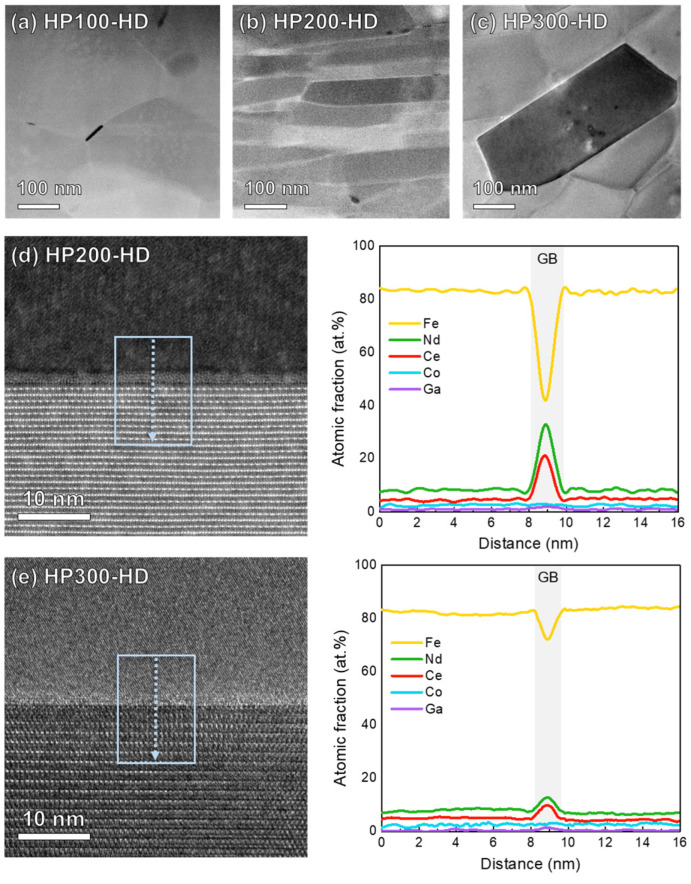
High-magnification ABF-STEM image of (**a**) HP100-HD, (**b**) HP200-HD, and (**c**) HP300-HD. Line concentration profiles obtained along the blue arrow direction indicated in the high-magnification HAADF-STEM images of grain boundaries in (**d**) HP200-HD and (**e**) HP300-HD, respectively.

Figure 5 presents high-magnification ABF-STEM images of the grain boundary of the hot-deformed magnets. In Figure 5a, misoriented grains with blurry boundaries are observed. Conversely, in the case of HP200-HD shown in Figure 5b, a uniformly distributed RE-rich grain boundary phase and well-aligned anisotropic grains are evident. In Figure 5c, coarsened grains exhibiting orientation distortion were observed due to non-uniform grain boundaries caused by the agglomerated h-RE_2_O_3_ secondary phase. Line-scan profiles obtained from the RE-rich grain boundary phase formed on the c-plane of the RE_2_Fe_14_B platelets of HP200-HD and HP300-HD are shown in Figure 5d,e. The RE concentration of the grain boundary phase in the HP200-HD (Nd+Ce = 54.02 at.%) is much higher than that of HP300-HD (Nd + Ce = 20.37 at.%). Generally, as the substituted content of Ce increases, the REFe_2_ secondary phase is formed, which consumes excess RE [21]. The formation of the REFe_2_ phase decreases the concentration of paramagnetic Nd and Ce in the RE-rich grain boundary phase in the final hot-deformed magnets, thereby suppressing the exchange decoupling between adjacent RE_2_Fe_14_B grains. In this study, both the HP200-HD and HP300-HD effectively suppressed the REFe_2_ secondary phase; however, in the case of HP300-HD, excessive formation and agglomeration of h-RE_2_O_3_ phase at triple junction resulted in decreased RE concentration at the grain boundaries. As a result, HP200-HD with high paramagnetic Nd and Ce in the RE-rich grain boundary phases effectively suppressed the exchange coupling and resulted in higher coercivity.

**Figure 6:**
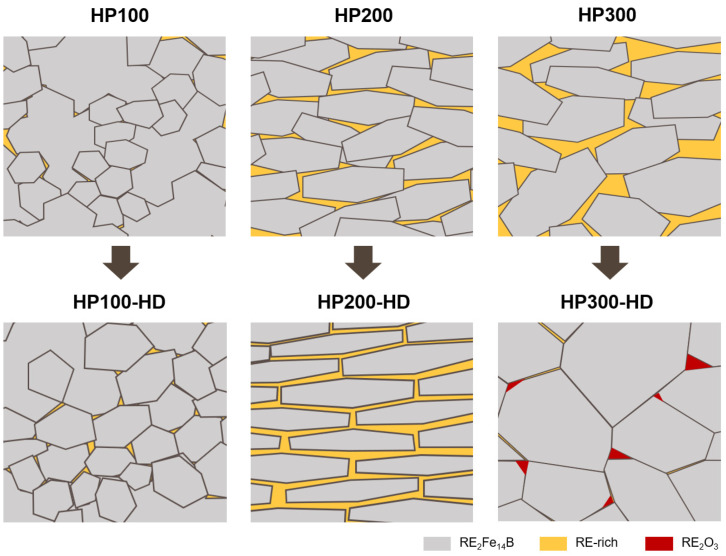
Schematic of microstructure evolution of the hot-deformed magnets based on the grains and grain boundaries of the precursors manufactured under different pressures.

Figure 6 is a schematic illustrating the microstructure at the flake boundary of hot-pressed magnets and hot-deformed magnets according to the pressure applied during the hot-pressing process. Under low-pressure condition (100 MPa) during the hot-pressing process, a porous sintered body with low density is formed, accompanied by limited precursor crystallization, leaving some areas in an amorphous state. Consequently, a subsequent hot deformation process leads to additional crystallization, leading to a notable reduction in anisotropy in the final hot-deformed magnet. This implies that when employing amorphous ribbon flakes, low-pressure conditions during hot pressing are unsuitable due to inadequate crystallization. Conversely, excessive pressure during hot pressing induces not only an uneven distribution of the RE-rich grain boundary phase but also encourages grain coarsening in regions with an oversupply of liquid phase [33]. The irregular and inefficiently aggregated distribution of the RE-rich grain boundary phase prompts the formation of h-RE_2_O_3_ with high oxygen content, significantly diminishing the wettability [17,38,39]. Consequently, grain boundary sliding is hindered, resulting in reduced anisotropy in the final hot-deformed magnets. Therefore, in the fabrication of Ce-substituted Nd-Fe-B hot deformed magnets using an amorphous precursor, the pressure applied during the hot-pressing process plays a pivotal role in both the crystallization of the precursor and the microstructure of the final hot-deformed magnets. In other words, when using an amorphous precursor, an appropriate pressure condition during the hot-pressing process suppresses the formation of the REFe_2_ secondary phase, enabling the production of hot-deformed magnets with well-aligned, uniform, and fine platelet-shaped grains.

## 4. Conclusions

In this study, we investigated the relationship between various pressures (100, 200, 300 MPa at 700 °C) during the hot-pressing process of Ce-substituted Nd-Ce-Fe-B hot-deformed magnets, based on amorphous precursors, the microstructure, and magnetic properties. Low-pressure conditions during hot-pressing resulted in the formation of porous, low-density sintered bodies, and induced low crystallization of the precursor. Consequently, the final hot-deformed magnets exhibited significantly reduced anisotropy during the hot deformation process. Conversely, excessive pressure during hot-pressing led to the formation of heterogeneous grain boundaries and promoted grain coarsening. Consequently, the final hot-deformed magnets exhibited an inefficient microstructure and lower magnetic properties. The appropriate pressure conditions (200 MPa) not only suppressed the formation of the REFe_2_ secondary phase but also facilitated appropriate crystallization and microstructure formation by using amorphous precursors. This resulted in obtaining hot-deformed magnets with well-aligned RE_2_Fe_14_B platelets along the c-axis and a high Nd/Ce-concentration paramagnetic grain boundary phase. As a result, we achieved high-performance and cost-effective Nd-Ce-Fe-B hot-deformed magnets with 40 wt.% of Nd substituted by Ce. Based on our research findings, we believe that maintaining appropriate pressure during the hot-pressing process is crucial for obtaining a uniformly distributed microstructure with fine platelet-shaped grains, which is essential for achieving high magnetic properties in hot-deformed magnets with high Ce contents.

## Figures and Tables

**Table 1:** Relative density and magnetic properties of the hot-pressed precursors and hot-deformed magnets.

Sample	Relative Density (%)	Magnetic Properties			Sample	Relative Density (%)	Magnetic Properties		
		*H*_ci_ (kOe)	*B*_r_ (kG)	(*BH*)_max_ (MGOe)			*H*_ci_ (kOe)	*B*_r_ (kG)	(*BH*)_max_ (MGOe)
HP100	89	9.6	5.9	6.1	HP100-HD	95	5.8	10.1	16
HP200	98	10.7	7.0	8.7	HP200-HD	99	8.9	12.2	31
HP300	98	10.8	7.2	9.2	HP300-HD	99	8.2	11.2	24

## Data Availability

The data presented in this research are available on request from the corresponding author (due to privacy).
